# Individual and contextual factors associated with community health workers’ performance in Nyanza Province, Kenya: a multilevel analysis

**DOI:** 10.1186/s12913-015-1117-4

**Published:** 2015-10-01

**Authors:** Yoshito Kawakatsu, Tomohiko Sugishita, Junya Tsutsui, Kennedy Oruenjo, Stephen Wakhule, Kennedy Kibosia, Eric Were, Sumihisa Honda

**Affiliations:** JICA SEMAH project, Kisumu, Kenya; Graduate School of Biomedical Sciences, Nagasaki University, 1-12-4 Sakamoto, Nagasaki, 852-8523 Japan; College of Social Sciences, Ritsumeikan University, Kyoto, Japan; Ministry of Public Health and Sanitation, Nairobi, Kenya

**Keywords:** Community health workers, Human resource, Performance, Multilevel analysis, Contextual factor, Rural area, Kenya

## Abstract

**Background:**

Several African and South Asian countries are currently investing in new cadres of community health workers (CHWs) as a major part of strategies aimed at reaching the Millennium Development Goals. However, one review concluded that community health workers did not consistently provide services likely to have substantial effects on health and that quality was usually poor. The objective of this research was to assess the CHWs’ performance in Western Kenya and describe determinants of that performance using a multilevel analysis of the two levels, individual and supervisor/community.

**Methods:**

This study conducted three surveys between August and September 2011 in Nyanza Province, Kenya. The participants of the three surveys were all 1,788 active CHWs, all their supervisors, and 2,560 randomly selected mothers who had children aged 12 to 23 months. CHW performance was generated by three indicators: reporting rate, health knowledge and household coverage. Multilevel analysis was performed to describe the determinants of that performance.

**Results:**

The significant factors associated with the CHWs’ performance were their marital status, educational level, the size of their household, their work experience, personal sanitation practice, number of supervisions received and the interaction between their supervisors’ better health knowledge and the number of supervisions.

**Conclusion:**

A high quality of routine supervisions is one of the key interventions in sustaining a CHW’s performance. In addition, decreasing the dropout rate of CHWs is important both for sustaining their performance and for avoiding the additional cost of replacing them. As for the selection criteria of new CHWs, good educational status, availability of supporters for household chores and good sanitation practices are all important in selecting CHWs who can maintain their high performance level.

## Background

The health systems in many countries are mostly fragmented, which hampers the scaling-up of essential interventions for maternal, newborn, and child health [1, 2]. One of the key challenges is an urgent need to develop and strengthen human health resources to deliver essential interventions [3–5]. The density of the health workforce is inversely associated with maternal, infant and under-fives’ mortality [6], and is more than ten times higher in Europe and North America than in sub-Saharan Africa. For this reason, the numbers of community health workers (CHWs) have been increasing recently, especially in low-income countries. Several African and South Asian countries are currently investing in new cadres of community health workers as a major part of strategies aimed at reaching the Millennium Development Goals [1]. However, one review concluded that community health workers did not consistently provide services that were likely to have substantial effects on health and that quality was usually unsatisfactory [7]. In addition, it suggested that all stakeholders need to focus on weak points in the functionality of the CHW program and ultimately the CHWs’ performance. The study in Kenya also showed that one of the significant factors positively influencing the full vaccination of children was a better CHW performance at the community level, but the variability of their performance was also reported [8].

The government of Kenya has begun implementing policies to improve health access, conducted by skilled health personnel. In 1994, the Kenya Health Policy Framework (KHPF) was developed to pursue the principles of the primary health care agenda. Based on these strategies, community health workers were established to conduct community-based health promotion activities. In 2006, the Community Health Strategy was developed and launched. Based on this strategy, CHWs and their supervisors, community health extension workers (CHEWs) in community units (CUs), were identified as Level One of the health system in Kenya [9, 10]. In 2010, the strategy was revised to improve CHW performance and make their work more effective. The main revised points were a stipend for CHWs and the allocation of the number of households to be covered per CHW. However, since the policy implementation was delayed due to physical and financial constraints, there was no influence from the revision at the time of this study.

CHWs were basically nominated from community members. At the time of this study, they were volunteers without financial incentives and received the ten days’ standard training developed by the Ministry of Health (MOH), Kenya. Some of them were given non-monetary incentives such as additional training, or identification such as T-shirts, depending on the partners and nongovernmental organizations (NGOs) supporting the MOH. Their main activities were door to door canvassing to teach health-related preventive methods and collect data from each household. There was no standard governmental monitoring and evaluation tool to measure a CHW’s performance except for their monthly report. In addition, CHEWs were identified as supervisors of CHWs. There were two types of CHEWs; one was a facility CHEW, who was a health worker, usually a nurse, working as a clinical professional in a health facility. The other was a community CHEW, who mainly worked with CHWs at the community level. Both kinds were trained using the standard training manual which contains the five modules [11]. In the manual, the components of supportive supervision are defined as follows: discussion with CHWs, performance evaluation, inspection of reporting tool, inspection of stocks of supplies, and development of an agreed action plan. Although the frequency of supportive supervision to be given to CHWs was not clearly stated, supervision on at least a monthly basis was implemented in the research area.

The objective of this research is to assess the CHWs’ performance in Nyanza Province, Kenya, and describe determinants of that performance using a multilevel analysis of the two levels, individual and supervisor/community.

## Methods

### Research site

This study was conducted in all 64 sub-locations covered by CUs that were established by May, 2011 in Siaya, Ugenya, Gem and Kisumu West districts, Kenya. The average population in each sub-location was around 5,000. The main ethnic group is Luo and their principal language is the Dholuo language.

### Study design and data collection

This study conducted three surveys between August and September 2011. The participants of the first survey were all 1,788 active CHWs in 64 CUs. The term “Active CHWs” was defined as CHWs who had been involved in any health-related community activity in the previous three months. Out of the 1,788 CHWs, 1,242 CHWs (69.4 %) who had no missing data were involved in the final analysis. After obtaining informed consent, the surveys were administered by self-administrative semi-structured questionnaires to assess the CHWs’ socio-economic characteristics, working style, job satisfaction and health knowledge. We asked open questions regarding immunization schedules, danger signs in pregnant women and neonates, risk factors of a dangerous pregnancy and so on to assess their health knowledge. Their answers were scored by the clinical officer, who had three years’ formal medical education, using the standard training manual developed by MOH Kenya as the gold standard. If the officer found answers she believed to be correct but were not in the manual, she consulted other clinical officer . The CHWs also reported the number of supportive supervisions given by their CHEWs within the last six months and their level of satisfaction in the CHEWs’ performance.

In addition, we conducted two other surveys at the same period focused on different study participants. The participants of the second survey were all CHW supervisors, so-called CHEWs, located within the research site. In this study we mainly utilized the data from field or community CHEWs, because they were direct supervisors of the CHWs. In the event that field CHEWs were not available, we used the data from facility CHEWs. These participants also answered a similar self-administrative semi-structured questionnaire. In addition, they submitted the reporting rate of the CHWs under their supervision. The participants of the third survey were mothers of children aged 12 to 23 months; this was to evaluate community level variables. After making a list of all the mothers, 40 in each sub-location were selected using random-sampling methods, giving a total of 2,560 mothers. They were targeted and asked, using an interviewer-administered questionnaire, to assess their socio-economic status, their health knowledge and their evaluation of the local CHW’s performance.

### Measurement

The dependent variable of this study was the level of each CHW’s performance, developed using three indicators: monthly reporting rate within the previous six months, health knowledge, and percentage of households covered by the CHW in the area (Table 1). We categorized each indicator into quintiles (0–4) to standardize the scores and generate the final integrated variable. The reporting rate within the last six months was classified as: no monthly reports submitted, 1 or 2 submitted, 3 submitted, 4 submitted, 5 or full reports submitted. Health knowledge was categorized as: lowest (<11 score), low (11–15.9), middle (16–19.9), high (20–25.9) or highest (26–34). This variable was also used as the performance indicator in a previous research article [12]. The coverage percentage of monthly visitation in each sub-location was grouped as: less than 20, 20–39, 40–59, 60–79 and 80–100 percent, which was also utilized in the research [5]. The correlation coefficient between the three indicators was low. The final evaluation of the CHW’s performance was the cumulative score of the three indicators, with the minimum and maximum score being 0 and 12 respectively. The highest score (12) indicated that the CHW had a good health knowledge, visited most of the households in their community and reported their monthly activities.

**Table 1:** Detail of sub-components of CHWs performance indicator

Score	Number of monthly reports in 6 months (0–6)		Health knowledge level (0–34)		% of community households covered (0-100 %)	
		N (%)		N (%)		N (%)
0	0	250 (20.1)	<11.0	159 (12.8)	<20	27 (2.2)
1	1-2	307 (24.7)	11.0-15.9	278 (22.4)	20-39	364 (29.3)
2	3	268 (21.6)	16.0-19.9	268 (21.6)	40-59	404 (32.5)
3	4	181 (14.6)	20.0-25.9	304 (24.5)	60-79	383 (30.8)
4	5-6	236 (19.0)	26.0-34.0	233 (18.8)	80-100	64 (5.2)

Twenty CHW, supervisor and community level variables were examined as independent variables. There were twelve individual variables in this study as follows. i) Gender was categorized as “male” and “female”. ii) Age was classified as “under 40” and “40 or older”, to achieve an equal distribution of participants. In addition, previous research showed that CHWs over 40 years old were associated with a significantly better performance [13]. iii) Marital status was categorized as “married” and “others”. This variable suggested support from partners, which may be a contributing factor to working effectively as a CHW. iv) Education level was grouped as: “no formal education”, “primary level” and “secondary level or higher”. Education was a factor in understanding health issues and knowing how to fill in the reporting tool. v) The number of household members was grouped as “fewer than six members” and “six or more members”. A larger household may equate to more support from family members for household chores. vi) The number of children under five was categorized as “zero” and “one or more”, as an indication of the CHW’s domestic duties in caring for and feeding children. vii) Family wealth index was generated using household materials, monthly salary and household assets, and categorized into quintiles as: lowest, low, middle, high and highest. viii) Sanitation practice was generated by assessing hand-washing practice, the availability of a toilet, hand-washing facility and dish rack, and the use of water treatment. Hand-washing practice was assessed by asking whether the CHWs washed their hands before preparing and eating food and after defecation. If they answered that they always washed their hands for all three occasions, we scored one; if not, zero. A toilet, hand-washing facility and dish rack scored one respectively, if available. In addition, if the CHWs’ households treated drinking water in any way, we scored one. After adding the scores of five indicators, we categorized them as “poor (0–3 points)”, “middle (4)” and “good sanitation practice (5)”. This variable reflected the CHWs’ level of health literacy and health consciousness. ix) The working experience of CHWs was classified as “less than four years” and “four or more years”. x) The number of households covered by CHWs was classified as “fewer than 21 households” and “21 households or more”. xi) Job satisfaction was assessed by the standard questionnaire [14], except for two sections regarding subjects such as Pay and Promotion, and classified into five categories: lowest, low, middle, high, and highest. xii) The frequency of supervisions received was grouped as: less than monthly, monthly and more frequently.

There were four variables describing supervisors’ characteristics and four community level variables as follows. xiii) CHEWs’ health knowledge was classified into tertiles as: low, middle, and high. xiv) Working experience as a CHEW was grouped as “less than 12 months” and “12 months or longer”. xv) The number of CUs managed by a CHEW was a continuous variable. xvi) CHEW job satisfaction, as assessed by the standard questionnaire, was classified into three categories as low, middle and high. As for community level variables, xvii) the average social capital in each sub-location was grouped as low, middle and high. xviii) The average community education level was grouped as low, middle and high. xix) Community health knowledge status was also grouped as low, middle and high. xx) Community wealth index was categorized as low, middle and high.

### Data storage and analysis

Data were double-entered after the data verification and stored using Epi info version 3.5. Statistical analysis was performed using Stata version 12 (Stata Corporation, TX, USA). The confidence level was set at 95 %. A descriptive statistic is shown in Table 2 to examine the characteristics of the sample.

**Table 2:** Individual, supervisor’s and community characteristics in Kisumu West, Siaya, Ugenya and Gem districts, Nyanza Province, Kenya

*Individual variables*	Number	%
Gender		
Female	1006	81.0
Male	236	19.0
Age group (years)		
<40	633	51.0
≥40	609	49.0
Marital status		
Single/Divorced/Widowed	19	1.5
Married	1223	98.5
Education level		
No education/dropped out of primary school	210	16.9
Primary level	462	37.2
Secondary level or higher	570	45.9
Number of household members		
Fewer than 6	497	40.0
6 or more	745	60.0
Number of children under five		
Fewer than 1	380	30.6
1 or more	862	69.4
Household wealth index		
Poorest	224	18.0
Poorer	291	23.4
Middle	268	21.6
Richer	207	16.7
Richest	252	20.3
Household sanitation practice		
Poor	169	13.6
Middle	362	29.2
Good	711	57.3
Years working as CHW		
Less than 4 years	630	50.7
4 years or more	612	49.3
Number of households covered by CHW		
Fewer than 21 households	745	60.0
21 or more	497	40.0
Job satisfaction		
Lowest	250	20.1
Low	261	21.0
Middle	233	18.8
High	251	20.2
Highest	247	19.9
Frequency of supervisions received		
Less than monthly	178	14.3
Monthly	908	73.0
More frequently	156	12.6
*Supervisor’s and community variables*	Number	%
CHEWs’ health knowledge		
Low	404	32.5
Middle	348	28.0
High	490	39.5
Months working as CHEW		
Less than 12 months	654	52.7
12 or more	588	47.3
Number of CUs covered (Mean, Std)	1.44	0.99
CHEW satisfaction score		
Low	254	20.5
Middle	735	59.2
High	253	20.4
Social capital		
Low	414	33.3
Middle	461	37.1
High	367	29.6
Community education level		
Low	365	29.4
Middle	564	45.4
High	313	25.2
Community health knowledge status		
Low	366	29.5
Middle	525	42.3
High	351	28.3
Community wealth		
Low	402	32.4
Middle	686	55.2
High	154	12.4

There were two levels in our data; level one was CHWs and level two was the supervisors and community level. In order to assess the roles of measured variables, as well as unmeasured factors at supervisor and community level, we used multilevel modeling in this paper. There were some assumptions that traditional regression methods were inappropriate methods for analyzing the nested datasets [15].

We estimated a multilevel model using *xtmixed* command in Stata [16]. We estimated four models: an empty model that contained no covariates, an individual model that contained individual variables, a community model that included all individual, supervisor and community variables, and a final model that contained all observed variables with the interaction between the number of supervisions received and CHEW health knowledge. The final model was selected based on the likelihood-ratio test and Akaike’s information criterion (AIC) [17].

Informed consent from all participants was obtained after full explanation of the study purpose and possible consequences. This research was approved by Great Lake University of Kisumu (GLUK) Ethical Review Committee (GERC) in Kenya.

## Results

Table 1 presents the detail of the sub-components which were utilized to generate the CHW performance score. Around 20 % had submitted their five or six monthly reports and got the highest health score. The average percentage of the number of households covered by CHWs in a community varied depending on the community. Only 5 % of them covered 80 to 100 % of the households in their community.

Table 2 shows the individual, supervisor and community level characteristics in our research site. Most (80 %) of the CHWs were female and half of them were over 40 years old. About 17 % had no experience of education or had dropped out from primary school. Around half of the CHWs had worked for four years or longer. About 13 % had been supervised more often than once a month by their supervisors. Around half of the CHEWs had worked less than 12 months and the mean of the CUs covered by each CHEW was 1.44. Almost a third (30 %) of them lived in a community with high social capital.

Table 3 describes the factors associated with the CHWs’ performance analyzed by a multi-level analysis. In null model (model 0), the intraclass correlation was 48 %. It indicated that the effect of the supervisor’s and community characteristics was significant enough that multilevel analysis was the appropriate analysis for this study. In the individual model (model 1) a middle (Coef.: 0.654, 95 % CI: 0.395-0.913) and high education status (Coef.: 0.934, 95 % CI: 0.679-1.190) were the positive significant factors associated with a CHW’s performance. If a CHW came from a larger household, their performance was significantly increased (Coef.: 0.201, 95 % CI: 0.0014-0.388). Longer work experience (Coef.: 0.339, 95 % CI: 0.129-0.549) and better sanitation practice (Coef.: 0.580, 95 % CI: 0.304-0.856) were also positive factors influencing their performance. It was noted that the number of supervisions received (Coef.: −0.713, 95 % CI: −1.083-0.344) negatively influenced the CHW’s performance. In the community model (model 2), while the same individual variables were significant, none of the supervisor’s and community level variables were significantly associated with the CHW’s performance. In the community model with the interaction (model 3), the intraclass correlation was 45 %. Married CHWs were more likely to give a higher performance (Coef.: 0.738, 95 % CI: 0.002-1.474). Middle (Coef.: 0.651, 95 % CI: 0.393-0.909) and high educational status (Coef.: 0.928, 95 % CI: 0.674-1.183) were positively associated with the CHWs’ performance. A higher number of household members (Coef.: 0.199 95 % CI: 0.013-0.386), longer work experience (Coef.: 0.327, 95 % CI: 0.118-0.537), and better sanitation practice (Coef.:0.616, 95 % CI: 0.340-0.331) were also positively associated with their performance. Monthly supervision (Coef.: −0.717, 95 % CI: −1.191--0.242) and more frequent supervision (Coef.: −1.230, 95 % CI: −1.863--0.597) were negative factors associated with CHWs. The interactions between the number of supervisions and the CHEW’s health knowledge were significantly associated with the CHW’s performance. The two interactions between better health knowledge and monthly supervision (Coef.: 1.140, 95 % CI: 0.488-1.791), and more frequent supervision (Coef.: 1.107, 95 % CI: 0.247-1.967) were positive influences on the CHWs performance, respectively.

**Table 3:** Individual, Supervisor’s and Community factors associated with CHWs’ performance in Kisumu West, Siaya, Ugenya and Gem districts, Nyanza Province, Kenya

	Model 0		Model 1		Model 2		Model 3	
*Individual variables*	Conf.	95 % CI.	Conf.	95 % CI.	Conf.	95 % CI.	Conf.	95 % CI.
Gender								
Female			Ref.		Ref		Ref.	
Male			−0.157	−0.384-0.070	−0.155	−0.381-0.072	−0.132	−0.358-0.095
Age group (years)								
<40			Ref.		Ref.		Ref.	
≥40			−0.176	−0.365-0.013	−0.174	−0.363-0.015	−0.158	−0.347-0.030
Marital status								
Single/Divorced/Widowed			Ref.		Ref.		Ref.	
Married			0.732	−0.006-1.470	0.727	−0.011-1.465	*0.738	0.002-1.474
Education level								
No education/primary school dropout			Ref.		Ref.		Ref.	
Primary level			***0.654	0.395-0.913	***0.655	0.396-0.914	***0.651	0.393-0.909
Secondary level or higher			***0.934	0.679-1.190	***0.933	0.678-1.189	***0.928	0.674-1.183
Number of household members								
Fewer than 6			Ref.		Ref.		Ref.	
6 or more			*0.201	0.014-0.388	*0.202	0.015-0.389	*0.199	0.013-0.386
Number of children under five								
Fewer than 1			Ref.		Ref.		Ref.	
1 or more			−0.182	−0.386-0.022	−0.189	−0.394-0.015	−0.186	−0.390-0.017
Household wealth index								
Poorest			Ref.		Ref.		Ref.	
Poorer			0.179	−0.094-0.452	0.180	−0.093-0.454	0.190	−0.082-0.463
Middle			0.055	−0.225-0.335	0.053	−0.227-0.333	0.055	−0.224-0.335
Richer			0.268	−0.034-0.564	0.261	−0.038-0.560	0.259	−0.039-0.557
Richest			0.169	−0.125-0.463	0.167	−0.128-0.461	0.163	−0.129-0.457
Household sanitation practice								
Poor			Ref.		Ref.		Ref.	
Middle			0.154	−0.131-0.439	0.150	−0.135-0.434	0.168	−0.117-0.452
Good			***0.580	0.304-0.856	***0.583	0.307-0.860	***0.616	0.340-0.331
Years working as CHWs								
Less than 4 years			Ref.		Ref.		Ref.	
4 years or more			**0.339	0.129-0.549	*0.330	0.119-0.540	**0.327	0.118-0.537
Number of households covered								
Less than 21 households			Ref.		Ref.		Ref.	
21 or more			0.159	−0.041-0.359	0.143	−0.058-0.345	0.131	−0.069-0.331
Job satisfaction								
Lowest			Ref.		Ref.		Ref.	
Low			0.110	−0.164-0.384	0.113	−0.161-0.387	0.103	−0.171-0.378
Middle			0.216	−0.066-0.498	0.218	−0.064-0.501	0.193	−0.089-0.475
High			−0.011	−0.295-0.272	−0.010	−0.295-0.274	−0.034	−0.318-0.250
Highest			−0.018	−0.300-0.264	−0.019	−0.301-0.264	−0.028	−0.310-0.255
Frequency of supervision received								
Less than monthly			Ref.		Ref.		Ref.	
Monthly			−0.156	−0.437-0.124	−0.150	−0.431-0.130	***−0.717	−1.191--0.242
More frequently			***−0.713	−1.083--0.344	***−0.709	−1.079--0.340	***−1.230	−1.863--0.597
*Supervisor’s and community variables*	Conf.	95 % CI.	Conf.	95 % CI.	Conf.	95 % CI.	Conf.	95 % CI.
CHEWs’ health knowledge								
Low					Ref.		Ref.	
Middle					0.096	−1.113-1.305	−0.280	−1.614-1.053
High					0.178	−0.717-1.072	−0.817	−1.868-0.233
Months working as CHEW								
Less than 12 months					Ref.		Ref.	
12 or more					−0.418	−1.301-0.466	−0.395	−1.270-0.480
Number of CUs covered					0.177	−0.320-0.674	0.172	−0.320-0.665
CHEW satisfaction score								
Low					Ref.		Ref.	
Middle					−0.835	−1.856-0.185	−0.811	−1.822-0.120
High					−0.796	−1.933-0.340	−0.753	−1.878-0.372
Social capital								
Low					Ref.		Ref.	
Middle					−0.097	−1.074-0.880	−0.103	−1.07-0.865
High					0.485	−0.540-1.510	0.469	−0.546-1.485
Community education level								
Low					Ref.		Ref.	
Middle					0.135	−0.732-1.001	0.130	−0.727-0.988
High					0.084	−1.072-1.239	0.087	−1.057-1.231
Community health knowledge status								
Low					Ref.		Ref.	
Middle					0.086	−0.732-1.001	0.107	−0.786-1.000
High					−0.184	−1.072-1.239	−0.183	−1.243-0.877
Community wealth								
Low					Ref.		Ref.	
Middle					0.656	−0.252-1.564	0.670	−0.229-1.569
High					0.973	−0.399-2.345	0.974	−0.384-2.333
Interaction								
(CHEWs’ knowledge and supervision)								
Low knowledge × Less than monthly							Ref.	
Middle knowledge × Monthly							0.473	−0.237-1.184
Middle knowledge × Frequent							0.326	−0.618-1.271
High knowledge × Monthly							**1.140	0.488-1.791
High knowledge × Frequent							*1.107	0.247-1.967
ICC	48.0		50.4		45.8		45.5	
AIC	4889		4780		4797		4793	
Log Likelihood (Likelihood-ratio test)	−2441		***-2366		-2360		*-2354	

*< 0.05, **< 0.01, ***< 0.001

## Discussion

This study described the performance of CHWs using three indicators: monthly reporting rate within the previous six months, health knowledge, and percentage of households covered by CHWs in Nyanza Province, Kenya. In addition, we identified the individual, supervisor and community level factors associated with CHW performance when analyzed by multilevel analysis. The performance of CHWs in this study was also varied, in the same way as other researchers have found [5, 13, 18]. According to our study, 20 % of CHWs had not submitted a monthly report within the previous six months. Although we checked their basic health knowledge, such as vaccination schedules and diarrhea prevention methods, 35 % of them could not get even half of the answers right. It is clear that more managerial and supportive interventions are needed to maintain a high performance of CHWs, although the government of Kenya has made considerable efforts to implement the community health strategy.

To develop an effective intervention, the following significant factors need to be given more attention. The significant factors associated with the CHWs’ performance were marital status, educational level, number of household members, longer work experience, better sanitation practice, number of supervisions received and the interactions between their supervisors’ better health knowledge and the number of supervisions.

Married CHWs gave a higher performance than others. It is also reported that marital status is significantly associated with the job satisfaction of primary health care workers in Nigeria [19]. This could be because they have more family members (not only their partner but also other relatives) to help with household duties. Having fewer household duties encourages CHWs to work more actively and reduces the dropout rate [20]. However, further study is needed to confirm the relationship between marital status and the amount of household duties. For the same reason, more household members at home were helpful in improving the CHW’s performance. One of the barriers preventing a good CHW performance was a heavy amount of household duties [20, 21].

High educational status was also a positive factor significantly associated with CHW performance. A high level of education would contribute to a high level of health knowledge, one of the performance indicators in this study. In addition, CHWs with a higher educational status would easily understand how to write and submit their monthly report. Other studies also reported the importance of CHWs’ educational status, including literacy level, in maintaining their high performance [20, 22–24]. This result suggests that one of the criteria in selecting a CHW should be a certain level of education. Although their capacity and time are limited to implementing various types of health services at the community level, CHWs with a high educational level perform better. CHWs who attended multi-purpose training gave a lower performance than CHWs who did not [18, 25, 26]. It would be difficult to add to the CHWs’ workload without additional incentives [27]. Thus it is important to select CHWs with a certain educational level and to focus on the limited number of cost-effective interventions.

Longer work experience was one of the positive factors in this study. If a CHW has a long work experience, they have had more opportunity to receive effective training, supervision and any incentives and to build a confidential relationship with community members. All these factors would positively influence their performance [5, 12, 28, 29]. Therefore, retention is one of the biggest concerns in sustaining effective community health activity, with influencing factors which have also been reported by various other studies [30–33]. One study in Kenya shows that the significant factors for CHW dropout are cultural background, inadequate support, poor selection criteria and the power difference between management staff and the CHWs [30].

Better sanitation practice, a positive influencing factor on CHW performance, would reflect their higher health awareness. It could indicate a greater interest in health and the motivation to learn about health-related issues and preventive methods. As one of the selection criteria, it would be better to check their sanitation practices, such as hand-washing and the availability of a toilet, although these behaviors would be changeable over time.

In this study, the number of supervisions had a negative influence on the CHWs’ performance. However, we argue that this result could indicate that supervisors monitor low-performing CHWs more frequently. This is because supervision is commonly one of the positive factors influencing CHW performance and a recommended activity [1, 12, 34, 35]. In addition, it is difficult to rationalize the negative impact of frequent supervision. In this study, the interactions between better supervisors’ health knowledge and the number of supervisions were also significantly associated with CHW performance. It means that although the number of supervisions increases among low-performing CHWs, when conducted by supervisors with better health knowledge they have a positive effect on the CHWs’ performance. In Table 4, we show the average performance score and each coefficient by the number of supervisions and the supervisor’s health knowledge. Compared with supervisions by those with low health knowledge, monitoring by those with higher knowledge had a positive effect on the CHWs’ performance. The high quality of supervision is one of the key factors in improving a CHW’s performance. Supervisors should have adequate health knowledge and conduct routine supervisions to sustain a high performance from the CHWs.

**Table 4:** Average of CHW’s performance and each coefficient by number of supervisions and supervisor’s health knowledge

			Supervisor’s health knowledge		
			Low	Middle	High
Number of Supervisions	Less than monthly	Ave	7.407	6.117	5.547
		(Coef.)	(Ref.)	(−0.280)	(−0.817)
	Monthly	Ave	6.099	6.168	6.780
		(Coef.)	(−0.717)	(−0.524)	(−0.394)
	More frequent	Ave	5.208	4.894	6.384
		(Coef.)	(−1.230)	(−1.184)	(−0.940)

Although we examined the various supervisor and community level factors, the large portion of ICC in model 3 still remained. It suggested that other factors which we did not examine in this study would be important in increasing the CHWs’ performance. According to other studies, social prestige [5, 21, 36], community support [5, 12, 21], and the training institute [13] are the important community level factors associated with CHW performance. Further research needs to take into account these community level variables.

## Limitations

This study is the first study on the performance of CHWs and influencing factors, including individual, supervisor, and community level variables, analyzed by multilevel analysis. Although our findings were informative and useful in developing effective interventions to sustain a CHW’s performance, there are several limitations in this study. Firstly, the performance indicators would need to be reconsidered. Although health knowledge and the number of household visits are relatively common performance indicators, health knowledge does not always improve health-related practices. A study shows that the practices of community health workers observed in a hospital setting were better than the practices observed in the community [37]. In addition, validation of the tools to evaluate CHW performance and other independent variables was not performed in this study. It would be better for further research to confirm the reliability of tools in a particular setting. This study showed just the association between the CHW’s performance and individual and contextual factors, not a causal relationship. A cohort study to determine such a causal relationship is needed to develop an effective intervention.

## Conclusion

This study described the CHWs’ performance, generated by reporting rate, health knowledge and average number of households they covered. In addition, individual, supervisor and community level factors influencing a CHW’s performance were also identified by multilevel analysis. According to our analysis, the significant factors were CHW’s marital status, education level, number of household members, longer work experience, better sanitation practice, number of supervisions received and the interactions between the supervisor’s better health knowledge and the number of supervisions. A high quality of routine supervision is one of the key interventions in sustaining a CHW’s performance. In addition, decreasing the dropout rate of CHWs is important both for sustaining their performance and for avoiding the additional cost of replacing them. As for the selection criteria for new CHWs, a good educational status, availability of supporters for household chores and appropriate sanitation practices are all important in selecting CHWs who can maintain their high performance level.
